# "The Hospital" Nursing Mirror

**Published:** 1900-12-01

**Authors:** 


### "The Hospital" Nursing Mirror.

Being the Nursing Section of "The Hospital."

[Contributions for this Section of "The Hospital" should be addressed to the Editor, "The Hospital" Nursing Mirror, 28 & 29 Southampton Street, Strand, London, W.C.]

### Notes on News from the Nursing World.

### THE LATE PRINCE CHRISTIAN VICTOR.

Some particulars are given in a letter to the matron of the Berkshire Hospital by Sister Sharp, of the Branch Yeomanry Hospital at Eastwood, Arcadia, Pretoria, of the nursing of the late much-lamented Prince Christian Victor. Miss Sharp, who was his night nurse, speaks in terms of high appreciation of her patient, who, even when he was extremely ill, expressed apprehension that he was "giving a lot of trouble." He also showed his interest in his nurse by asking where she was trained, where she came from, and how long she would be on night duty. The Prince had a room to himself, and in addition to the sister in attendance on him night and day, there were two special orderlies. We think it is a pity that such details should have been communicated to the daily press; but no doubt many people besides members of the Royal Family will be relieved to be assured that nothing which could have been effected by careful and devoted nursing was left undone.

### THE HOSPITALS COMMISSION REPORT.

We are able to give an unqualified contradiction to some rumours about the Hospitals Commission Report which have gained a certain amount of credence, in spite of the fact that they were obviously merely conjectural. The draft report of the Commission has not been completed, and there is not the remotest likelihood of the report itself seeing daylight this year, much less of its being presented to Parliament next week. It follows that the talk about it "having been decided not to replace any of the male orderlies by female nurses in any of the military hospitals" is idle chatter. Whatever conclusion the Commission may arrive at, it is only fair that no attention should be given to irresponsible gossip, and that nurses and the public should not attempt to pronounce judgment in the matter until they know, not only the nature of the recommendations of Lord Justice Romer and his colleagues, but also the grounds upon which they are based.

### ROYAL BRITISH NURSES' ASSOCIATION.

The thirteenth annual conversazione of the Royal British Nurses' Association will be held on Tuesday next, at 8 p.m., in Westminster Town Hall. The programme includes recitations by Canon Fleming, songs by Mr. Sterling Mackinlay and Mr. Stepney Rawson, madrigals by Mr. and Mrs. Rawson, Miss Lena Law, and Mr. Sterling Mackinlay, musical sketches by Mr. R. H. Manning, and banjo sketch by Mr. James Dunn, of the Palace Theatre. During the whole of the evening the electrophone, connecting the leading London theatres and concert halls, will be available, and the Æolian Ladies' Orchestra will play selections of music. Tickets can be obtained from the secretary, 17 Old Cavendish Street, W.

### AN INCIDENT AT WYNBERG.

A nurse at Wynberg found that some extra milk ordered by the medical officer for the night had been consumed, though all the patients denied the theft. As a punishment they were told that they would not be allowed any stout during the day. Two hours afterwards the enclosed piece of poetry was presented to the nurse by one of the culprit "Tommies":—

Who Drank the Milk?

My dear comrades in a thirsty plight,
Be careful what you do another night;
If you drink the milk, they'll find you out,
Then you must surely lose your stout.
But, my friends, I do not think
The milk was ever there to drink;
And this through my mind does throng,
We lost our stout for another's wrong.
And if they want revenge for the milk, I say
They must take the orderly's dinner away;
For the cook could have only given him half,
Or else he was the thirsty calf
Who drank the extra milk.

A Thirsty Patient.

### CHRISTMAS PREPARATIONS.

The holly bough "is not considered surgically correct," said the matron of Charing Cross Hospital to our representative; "the germ theory has put a stop to it." And this sentiment was echoed by the matrons of Westminster, where a few evergreens are put up the day before, and taken down the day after Christmas Day; King's College, where a few wards are decorated, "if the surgeons do not object"; the Middlesex, where the lady superintendent described the use of evergreens as "most insanitary"; the Royal Free and University College Hospital, where they are condemned for the same reason. But there will be plenty of gaiety for the patients, as usual, though it is possible that it may take a milder form this year on account of the war. The patients at King's will have their friends to visit them during the afternoon of Christmas Day; Christmas trees and bran tubs will be provided for the children at Westminster, and entertainments will be given by subscribers and friends at Charing Cross. "We shall generally devote ourselves to the patients," say the matrons, "and make Christmas time pass pleasantly for them; but there will be nothing out of the common—nothing worth special notice." The Middlesex will have its usual big Christmas tree in the board room for the "babies," and another tree on New Year's Day for the little boys, and there will be, as heretofore, a good Christmas dinner in all the wards, when the tables will have plenty of flowers and plants. Turkeys and plum puddings will be provided at King's.

### AS BEFORE AT GUY'S.

Of course at Guy's the old and popular plan of hanging festoons of evergreens with scarlet berries across the wards will be followed, and if there is any diminution in the decorations this Christmas it will be on account of the new wards. "We may not decorate these, perhaps," said the matron, "because they look very bright and pretty already. There will be a Christmas tree in each ward, lanterns, and plenty to entertain the patients."

### THE FAMILY PARTY AT THE ROYAL FREE.

"After long experience," said the matron of the Royal Free Hospital, "we have evolved the Family Party as the most effective form of Christmas entertainment. If the patients are in bed, the party will be held by the bedside; if well enough to be up but not to go home, the patients and their friends will have tea at long tables in the wards, decorated profusely with flowers and plants; and there will be tea and cakes and oranges and buns for everyone, and a Christmas tree and presents for the children. We cannot give the grown-up guests presents, but we usually manage for all the visiting children to have something." It is the next best thing, from the patients' point of view, to going home for Christmas, Miss Wedgewood thinks. "If it is the mother who is in hospital," she said, "it is the father who comes and brings the children; if the father, then the mother comes, and so on. Then on another day we have an entertainment by the students, who go through the wards with lanterns and sing carols: they are very kind in making Christmas a pleasant time for the patients.

### NO CHRISTMAS DECORATIONS AT UNIVERSITY COLLEGE HOSPITAL.

"At University College Hospital," said the matron, "we shall not have time to consider Christmas decorations, and I hope they will not be allowed in the new wing, as they are most unwholesome. Our time is very much taken up with preparations in connection with the opening ceremony, which will, we hope, take place about December 22nd, just a few days before Christmas. We shall be very glad to get our nurses into the new building: they are at present living in seven houses outside, and it makes discipline very difficult. So you see that we are too busy to think of much besides our new accommodation."

### NURSING THE SUFFERERS FROM BEER-DRINKING.

The extraordinary epidemic at Manchester due, as it has been ascertained, to arsenical poisoning, the poison being contained in beer, has entailed an immense amount of extra work upon the nurses at Crumpsall Workhouse Infirmary. It appears that the victims are by no means confined to Manchester, and reports from Salford, Birkenhead, Liverpool, Stourbridge, Worcester, and parts of Yorkshire, point to the fact that serious illness has been produced in many other cases from the same cause, though previously attributed to something else. There is no difficulty in the nursing, which is the same as that of ordinary peripheral neuritis cases plus the skin eruptions. Apart from the number of deaths which have been traced in Salford and Manchester alone to the poisonous ingredient found in the local beer, the sufferings of many of the patients have been and are severe, and they, of course, require constant and careful attention.

### THE RESULT OF NURSE DOWLING'S ASSERTIONS.

The communication of Miss Dowling, which we published on November 17th, has had the result of inundating us with correspondence from nurses of all grades in all parts of the country. Last week we gave a selection, and to-day several further letters will be found in another part of the paper. With a single exception, it will be observed, every correspondent, whether a matron or a probationer, denies the accuracy of Miss Dowling's allegations, and defends both hospital managers and matrons with enthusiasm. Even "A. P.", who confesses that her experience has been small, is more concerned to point out that she is laying down her life for the patients whom she assists in nursing, than to endorse Miss Dowling's accusations. But her letter is the only one we have received which does not tend to show, in a more or less convincing manner, the groundlessness of an indictment formulated, as we think, when the author was smarting under defeat and disappointment.

### A SUPERINTENDENT NURSE APPEALS TO THE LOCAL GOVERNMENT BOARD.

The East Preston Guardians having demanded the resignation of Miss Rogers, the superintendent nurse, she has appealed to the Local Government Board to intervene. She maintains that ever since her appointment, fourteen months ago, she has done her utmost to carry out the duties of her office with conscientiousness and to the best of her ability, and, in asking for an inquiry, she alleges that she has been hampered in her duties by the master and matron, and by the want of support by the Board, whom she accuses of having vented upon her their annoyance at having to appoint, by the direction of the Local Government Board, a superintendent nurse. The reply of the Guardians has been sent to Whitehall, and is in effect the report of the Nursing Committee which prompted their decision. They affirm that Miss Rogers made some complaints so trivial as to call for no remark, and put forward others with an evident desire to be allowed full control in the infirmary. "This they could not countenance for a moment." But the whole drift of the report is to cast upon the superintendent nurse the onus of the blame for an admittedly unsatisfactory state of things in the infirmary. We offer no opinion on the case at the present stage. As Miss Rogers has held several appointments of importance, and her professional reputation is at stake, an inquiry is of obvious importance to her; but it is of still greater importance in the interests of the patients and the public that any doubts about the cause of the unsatisfactory state of affairs at East Preston Workhouse Infirmary should be cleared up.

### IN THE NAME OF SISTER DORA.

The name of Sister Dora will never fade away from the memory of the people of Walsall, the town in which she lived and laboured and died. At a bazaar in aid of the Walsall and District Hospital, opened by the Duchess of Sutherland, the Duchess herself alluded to the work of that devoted woman, and justly said it would be ill-fitting if they let the footsteps of the great who had passed away for ever be effaced. Sir Arthur Hayter, M.P., also said that "it would be a crying shame if in the scenes of Sister Dora's labours there were any slackness in providing accommodation for the hospital and for the nurses." The latter is badly needed, and the support given to the bazaar affords promise that the total sum of £6,000 required for the work will soon be subscribed. Contributions of upwards of £4,000 to the building fund were announced by the Mayor.

### INCREASE OF NURSES AT BELFAST INFIRMARY.

In spite of an attempt to defeat the recommendations for the increase of the nursing staff at Belfast Infirmary, the Board of Guardians have given effect to them, and in future there will be twenty nurses attached to the institution, two of whom only are not new. How essential it was to lose no time in acting upon the recommendations of the committee may be judged by the fact that under the old régime, within four or five weeks six nurses had resigned, four of them being charge nurses, one a probationer who left on the score of health, and the sixth a temporary nurse. Mr. Oswald wanted the Board to appoint only six nurses in place of the six resigned, but the general feeling, happily, was that the way to keep good nurses was to furnish an adequate supply for the performance of the duties without hardship to any. Moreover, the nurses of the Belfast Infirmary are shortly to have a new home on modern lines.

### A TRIBUTE FROM THE WORKING MEN OF TAUNTON.

It is very pleasant to learn that the working men of Taunton have decided to erect a tombstone on the grave of the late Miss Fisher, in memory of her devoted and self-sacrificing labours amongst the poor of the town during the many years she was associated with the District Nursing Association as hon. superintendent and hon. secretary. This is a striking proof of the value attached to the work of the Nursing Association, and those who have benefited by it are showing their gratitude in a very graceful manner. Taunton is quick to recognise the self-denying labours of those engaged in nursing, for not long ago a stained-glass window was put in St. Andrew's Church in memory of Nurse Sage who was formerly connected with the Taunton Nursing Association, and volunteering for service in South Africa with the Welsh Hospital Corps, died soon after reaching Bloemfontein. A window is now to be put in the same church as a memorial of Miss Fisher.

### A SERIOUS QUESTION FOR THE LOCAL GOVERNMENT BOARD.

We direct the attention of the Local Government Board to our account of the proceedings of the Croydon Board of Guardians on Tuesday. It will be seen that a majority of the Guardians have adopted a report of their own infirmary committee which, if acted upon, will deprive the matron of all authority, and reduce her to the position of a cipher, and this solely because, in the exercise of her discretion, she refused to sign the certificates of three probationers. We hope that the authorities at Whitehall, whom the chairman of the Board of Guardians accuses of "sitting on the fence," will come down sharply on the right side, and insist that, in the interests of nurses and of the public, Miss Julian shall have the power to which she is entitled restored to her. If such a precedent as it is sought to establish by the Croydon Guardians is once set up, the value of all certificates from workhouse infirmaries will be practically *nil*, and the difficulty of obtaining nurses, already very great, will be enormously increased.

### YORK WORKHOUSE INFIRMARY.

At a meeting of the York Board of Guardians a letter from Dr. Shann, the medical officer, urging the need of more nurses was read. Mr. Shaw made the remarkable suggestion that the Press should not publish the letter "at present." We are glad to say that the suggestion was ignored, for Dr. Shann's communication is one of general interest. He considers that there should be two night nurses on the female side of the infirmary where there is now one; that on the male side there should be one male attendant to attend to the imbecile and epileptic wards, and a female night attendant for the infirmary wards on the male side; and that an extra nurse is required for day work on the female side. He also proposes that the night and day work should be more evenly divided as to nurses in attendance, and points out that under existing conditions the day nurses, "with much more actual work," have much longer hours. His letter has been referred to the House Committee to deal with, and in the interests of both patients and nurses at York Workhouse Infirmary, we trust that the change he advises may be made without delay.

### THE KILBURN SISTERS.

Lady Frederick Bruce, of Everton Grange, Lymington, sends us a paper headed "The Kilburn Sisters," which, she intimates, explains why the Sisters of the Community of St. Peter object to being known as "Kilburn Sisters." We need not enter into any of the personal questions which are raised in the pamphlet, but we quite understand that the Sisters of the Community of St. Peter do not wish to be identified with an organisation which declines to submit to episcopal authority. So far as nursing is concerned, this point is, of course, beside the issue, and there may be Kilburn Sisters, no less than Sisters of the Community of St. Peter, who have done good work in nursing.

### A USEFUL ORGANISATION.

The first general annual meeting of the Royal London Ophthalmic Hospital Guild was held at the Hospital last week. The Lady Mayoress has consented to become President of the Guild. Lady Avebury, Lady Russell Reynolds, and Mrs. John Tweedy have been elected Vice-Presidents. The Guild has supplied the Hospital with garments for the use of patients in the children's ward and special garments for the patients to wear in the operating theatre. It has also given a large quantity of house linen to the Hospital during the past year.

### OUR CHRISTMAS DISTRIBUTION.

We have received parcels of useful articles from Miss Annie Burt, Post Office, Lelant, Cornwall, and Miss Kate Saczkovie, Mitcheldean, Gloucestershire.

### SHORT ITEMS.

The Victoria Central Hospital, Liscard, Cheshire, of which Miss Bailie has been appointed matron, will be open for the admission of patients in January. A most successful bazaar has just been held in the hospital at which over £5,600 was raised in aid of the furnishing funds.—The ss. *Dilwara*, which has lately arrived, had on board six army reserve nurses and two nurses of the Irish Hospital, sisters Macdonnell and Walker.—An entertainment of an excellent character was given to the in-patients of the Cancer Hospital, Fulham Road, on Thursday evening, by the Social Dramatic Company under the direction of Mr. Reginald H. Lindley. At the conclusion, Dr. Herbert Snow proposed a very hearty vote of thanks to all who had so kindly given their services to amuse the patients.—"All convalescent," is the report given by nursing Sister Mark (A.N.S.) of the patients on board the *Orcana*. The voyage was uneventful, and Miss Mark says "nothing of any interest occurred this time, we had a good, quick passage." The *Orcana* sails again for Durban about the beginning of December.

### Lectures on Medicine to Nurses.

By H. A. LATIMER, M.D. (Dunelm), M.R.C.S. Eng., L.S.A.Lond.; Consulting Surgeon, Swansea Hospital; Past President of the South Wales and Monmouthshire Branch of Brit. Med. Assoc.; Past President of the Swansea Medical Society; Hon. Life Member of the St. John Ambulance Association, &c.

CAUSES OF FEVERISHNESS.—*Continued.*

I STOPPED my last lecture at a point where I had just told you that the feverish process known as catarrh was the result of any congested state of a mucous membrane. Now, inasmuch as many causes are at work in irritating mucous membranes, it is not difficult to see what a fertile cause of feverish attacks this proclivity to catarrh is. Let us spend a few moments in reviewing possible causes of mucous membrane irritation.

Apart from the fact that every mucous membrane is liable to an especial irritation from the disturbing qualities of matter brought in contact with it, it has to be remembered that general disturbing agents are at work at all times by which they may be affected. Thus, taking the air passages as an example, any part of this mucous tract, from the nose to the finest divisions of the bronchial tubes, may be irritated by such widely-differing agents as air which is too hot or is too cold, by dust, by the presence within the blood circulating there of the poisons of the specific fevers (as, for instance, the poisons of measles and of typhoid fever), by the process of chill elsewhere, at the extremities of the body, whereby the contracted blood-vessels at the chilled parts contract, and internal parts are flushed with more than their normal quantity of that fluid; and by impurities, present in the breathed-in air, which have been derived from crowded rooms where gas or too many people for the cubic space of the place have exhausted the oxygen, and have replaced this pure agent by impurities resulting from processes for the manufacture of coal, and from the skins and lungs of the people present.

The mucous membrane of the food passages is liable to suffer from a hundred differing irritations, so diverse in character that one might draw up quite an exhaustive catalogue of them were one minded to do so, and were one wishful of exhausting your patience and of defeating the very object of these lectures, which is to interest without fatiguing you. Suffice it to say that decayed teeth may set up congestion of the adjoining gums and tongue, that improper foods and drinks may do the same in the stomach and intestinal canals, and that chilled surfaces of the abdomen, or of limbs, may also set up catarrh of the internal mucous membrane, either through reflex action or by direct flushings and congestion of it by the diverted blood from the skin. And so on through the whole catalogue of mucous membranes.

Another great cause of simple feverish state is, undoubtedly, the disturbance to the nervous centres arising from the supply to them of a blood which is of impure quality. Such impure blood circulates through the vessels when, from any severe disorder of the excreting organs of the body, poisonous matters which it is their duty to convey out of the system are retained there. You know that by means of the urinary organs those highly poisonous substances, urea and uric acid, are separated from the blood and cast out of the body: these substances are the ashes of the tissues of the body which have been burnt up by oxidation in the wear and tear of life. They are intensely poisonous, and health, nay life itself, can only be maintained by their being carried away in the urine. If they are manufactured in excess, or if they are not removed from the blood in proper quantities, disease results. And by temporary derangements of the skin and kidneys the normal excretion may be interfered with, and fever declares itself. I take it that, putting aside those cases of febricula which have their origin in nerve disturbances, such as teething, the great number are caused by the retention in the blood of some peccant material which should have been removed from it by one or other of the excretory organs. Such wrongful matters may be retained when the lungs are prevented by any cause from exchanging the poison carbonic acid for the life-giving gas oxygen; when the skin cannot keep up an insensible perspiration and act as an accessory breathing organ; when the liver is unable to perform its offices of destroying old effete blood-cells and of manufacturing bile; when, from the existence of intestinal catarrh, the various glandular digestive actions are prevented from taking place; and when, from constipation, excrementitious matters are retained within the bowels and re-absorption of some of them is effected; and when, as I have before said, by derangement of the kidneys and urinary organs, the poisons which it is their duty to hurry out of the system cannot escape from it and accumulate in the blood.

The point to be noted is that in febricula the operating cause of the attack is a transient one and the resulting fever is short-lived; were it otherwise the affection would pass beyond this simple condition and we should be confronted with appreciable inflammations of organs. Regarded from this point of view you will see that the bundle of symptoms which we call by the name "fever" are simply directions to us to seek for some operating cause which is behind them, and that if that cause is dealt with, by a righting of what is acting wrongly, we are working in the best manner for disposing of the fever. Herein lies the difference between a scientific and a quack system of medicine. A quack system deals with symptoms, levelling medicines at them, and not seeking to attack the prime cause of those cries of suffering organs; a scientific system aims at finding out causes of symptoms and at treating such causes with a view to abolishing symptoms. I think that in this difference of mental attitudes towards disease lies one reason why the quack has such a good innings with mankind. People generally know little of physiology and less still of pathology, and are unable and unwilling to grasp truths about the prime causes of disease. Moreover, knowing that in many illnesses they have themselves to thank for their troubles by their disregard of the laws of health, they are unwilling to attack disease from this side or to recognise as the origin of their suffering habits which they cling to. The quack who won't bother them with too much inquiry on such matters, but who will level a drug at each prominent symptom, with a confident promise of its removing the same, will have a big following. And the empirically minded man will often effect cures when the man more deeply versed in knowledge of the nature of disease will fail to do so, for the arts of diagnosis and of treatment of disease by no means always go together. Happy is the man who has the practical application of theoretical knowledge! It is by no means rare to meet with men of the highest reputation for diagnosing diseases who are almost useless as practical physicians. They often fail to cure by not cultivating this side of the question, when a less learned but more practical man blunders to success by a blind treatment of symptoms. What the symptoms and the treatment of febricula are I will tell you in my next lecture.

(*To be continued.*)

### Two Pictures from Pretoria.

By An Army Sister.

### GLORY.

The annexation of the Transvaal is an accomplished fact. I was among those who had the pleasure of witnessing the formal ceremony on Thursday, October 25th. We were most fortunate in having an invitation to the Raadzaal from the numerous windows of which an admirable view of the whole proceedings was to be had. The Market Square, in the centre of which, oddly enough, stands the Dutch Reformed Church, was the chosen spot for the historic scene. Here, too, is the *pedestal* for a monument of Mr. Kruger. It stands in suggestive incompletion, and one cannot but hope that nothing will be erected on it to spoil the moral it silently teaches. The Square was lined with soldiers, a detachment of the Coldstream Guards standing immediately behind the flagstaff so soon to proclaim freedom and justice to the land over which its flag flies. There was a fair sprinkling of Dutch or Boers among the English who thronged the Square. One wondered what had brought them. Idle curiosity perhaps, or possibly deeper feelings.

Punctually at 4 P.M. Lord Roberts and his staff rode into the Square, the band struck up "God Save the Queen," and the Royal Standard, worked by some local talent, was run up the flagstaff. The proclamation, of which, unfortunately, we could not catch many words, was read by the Provost-Marshal, Major Poore, alone on horseback, in the centre of the Square. The band again played the National Anthem, which was followed by three ringing cheers, led by Lord Roberts. We were not merely to have the pleasure of joining in and witnessing this ceremony. Fortune was kind and provided—at any rate to me—the unique occasion of the presentation of seven Victoria Crosses. Surely it was a proud day for the men—six officers and one corporal of the Gordons —when marching up in line to the centre of the Square, where on a small table lay covered with the Union Jack, the much coveted medals of distinction. They had their Crosses pinned to their breasts by Lord Roberts, the soldier's idol, who shook hands with each recipient. A march past then took place, numbering between 6,000 and 7,000 soldiers—among them the Scotch Fusiliers, who were in Pretoria in 1881, when the British flag was pulled down and the Transvaal hoisted in its place. It seemed as if there were no end to the oncoming lines of khaki. A 6-inch gun drawn by fourteen yoke of oxen—the front couple led, as usual, by a Kaffir—caused a good deal of amusement, for he had hung, instead of wearing, a pair of ostentatiously new boots round his neck, and marched barefoot and erect in front of his gun. When all the artillery, cavalry, and infantry had marched past, Lord Roberts addressed the Household Cavalry and other mounted men, who were then drawn up in full force in the Square. Very unfortunately, we could not catch what was said. One thing we thoroughly enjoyed was the first-rate view we had of the splendid horses of the Staff officers, reminding one just a little of the Military Tournament. Finally, Lord Roberts rode off amid an accompaniment of cheers, and we went home, feeling glad and proud that we were English people, and speculating as to the effect produced on the Boer mind by the fine display we had just witnessed.

### GLOOM.

A week after and Pretoria was *en fête* for the ceremony of the Annexation of the Transvaal, flags were flying half-mast, and the mournful minute-guns forced on one's attention yet one more sacrifice exacted by this sad war in South Africa. The occasion was the funeral of Prince Christian Victor, grandson of the Queen. He died from enteric—that fell disease which spares no rank, and seems to defy every effort of nursing. At 10 in the morning the guns began to fire when the sad procession left the Yeomanry Hospital, where he died: the whole route, covering, roughly, four miles, was lined with troops, and groups of people assembled silently along the roads to watch what was indeed a fine and touching sight—a military funeral for a distinguished officer. Rather more than half way to the cemetery is the Cathedral, where a short service was held. Far away in the distance one could hear music, the muffled drums and the minute-guns, gradually the solemn notes of Chopin's "Marche Funèbre" became distinguishable, growing in fulness and beauty as the band and the sad cortège approached. While waiting in the Cathedral, through the open door we had a good view of the procession as it passed. Two guns drawn by six horses each formed the front, followed by a large body of Mounted Infantry with rifles at salute. These were succeeded by the Rifle Brigade and then the Grenadier Guards, marching so slowly with arms reversed—a most impressive sight—then a large body of orderlies; and lastly, escorting the gun carriage on which the coffin, shrouded in the Union Jack, lay, with wreaths of white flowers on the top, came a large detachment of the Coldstreams, and, pathetic sight, the Prince's beautiful black horse, saddled and riderless. The band now struck the grand and mournful notes of the "Dead March" in *Saul* as the coffin, borne by 12 soldiers and escorted by eight pall bearers of high rank, entered the Cathedral. Carried slowly up the aisle it was placed on a raised daïs, covered with a violet velvet pall, and partly surrounded by palms in the chancel, while a short service was held. Lord Roberts, Lord Kitchener, and many other generals and officers were present, as well as the chief mourners. Those nurses who were able also came to pay a last mark of respect. Then to the music of the band once more the procession wended its way to the cemetery. The minute-guns had never ceased firing, and did not do so till all was over. So among the many brave men who have fallen by disease, wounds, or battle on African soil, and found a last resting-place there, we must include a Royal Prince.

### Presentations.

Eastville Hospital, Bristol.—Miss H. Hannath who has been for two years superintendent at the Eastville Workhouse Hospital, was presented on leaving, by the sisters and nurses, with a gold curb bracelet and framed photograph of the nursing staff in token of their appreciation and the high esteem in which she is held by them all. Among other presents which Miss Hannath received was a silver sugar bowl and sifter from some of the officers of the House.

### Where to Go.

Exhibition of Old Embroideries, &c. — Messrs. Debenham and Freebody, Wigmore Street, have arranged a charming little exhibition of old embroideries, brocades samplers, quaint needlework pictures, and ancient fabrics that will repay a visit. Although the articles are for sale, visitors are welcomed and are not pressed to purchase. The exhibit contains articles to suit all purses, and the whole formed a famous collection purchased from Howell and James.

### "The Hospital" Convalescent Fund.

We have to acknowledge with many thanks the following contributions:—Miss A. Burn-Bailey, 5s.; Nurse E. Jarman, 2s. 6d.

### The Nurse in Hot Climates.

By EDWARD HENDERSON, M.D., F.R.C.S. Edin., late Surgeon, General Hospital, Shanghai.

(*Continued from page* 105.)

*Prophylaxis.*—It is certainly one of the nurses' duties to guard, as far as lies in her power, against the spread of disease from the sick to the healthy. Even when full instructions regarding the proper measures to be taken in any particular case are given by the doctor, success will ultimately depend on the careful and intelligent manner in which these are carried out by the nurse and her assistants.

The nurse must have some clear views regarding the probable channels through which the contagia of the new diseases she is brought in contact with enter the body, before she can hope intelligently to carry out precautionary measures to guard against their invasion.

In cholera and the epidemic forms of dysentery and diarrhœa, from the mere fact that the early symptoms of these diseases are from the first manifested in connection with the organs concerned in the digestion and assimilation of food, while the principal lesions seen in the body after death are found in the intestines, it is reasonable to suppose that infection is due to a contagium which has entered by the mouth as a swallowed poison. Nearly the same may be said of enteric fever, a disease with which the nurse's previous training in England has already made her more or less familiar. The micro-organisms on which cholera and typhoid depend have been isolated, and positive proof obtained by experiments on the lower animals that these diseases can be introduced through the alimentary canal.

The poison of plague is believed to enter the body either through the skin or the mucous membrane. In the skin a breach of surface is probably always needed for its introduction, but in the mucous membrane the necessity for this is less apparent. In bubonic cases the position of the glands first affected has been thought by some to indicate the probable situation of the primary lesion: thus, when the groin glands are the first to suffer, the lower extremity is suspected; when the cervical glands take precedence, the disease is supposed to have entered by the tonsil. The specific organism which is the cause of plague has been isolated, and experimental proof given that in the lower animals, besides inoculation, the virus can be introduced in food, and may also be conveyed through the nostrils without any apparent loss of continuity in the lining of these cavities. The specific bacillus of plague is widely disseminated in the bodies of the patients, making the disease a highly contagious one in many ways: the blood contains it, it is found in the contents of buboes, occurs in the urine and the fæces, and exists largely in the sputum of pneumonic cases. The bacillus has not yet been found in the air surrounding the patients, but this cannot be regarded as excluding the air passages as possible channels for its introduction. The occurrence of cases in which pneumonia is from the first the main feature of the disease, favours the view that the poison of plague is one which may sometimes be inhaled.

As already pointed out, the blood parasite which is the cause of malarial fever in man is conveyed by the mosquito, and, so far as our present knowledge goes, this is the only way in which that disease is transmitted.

In cases in which infection is probably the result of a swallowed poison, the attention of the nurse will naturally be first directed to the possible contamination of the food she uses and gives to her patients; and along with this, personal cleanliness, especially the washing of soiled hands, will suggest itself as of scarcely less importance. Should infection by the air passages be the danger which threatens, the nurse will give special attention to the ventilation of wards and sick-rooms, avoid prolonged exposure as far as she is able, and make use of an antiseptic mouth-wash or gargle. When the virus is one which can be inoculated, surface wounds, especially those situated on the hands, must receive immediate attention. When possible channels are many, the precautions taken must be multiplied in proportion.

*Contamination of Food and Drink, &c.*—This is the danger chiefly to be apprehended in cholera, which may be taken to represent that class of diseases in which infection is believed to enter the body by the alimentary canal as a swallowed poison. When night soil is removed by hand labour, as it is everywhere in China, and, with the exception of a few large towns, nearly everywhere in India, the danger of direct contamination may be increased. The night soil removed in this way is of necessity temporarily stored in the compound or basement of the hospital or dwelling-house in which it has accumulated, to await its final removal from the neighbourhood in carts or buckets; and, when flies are numerous, it is evident that a dangerous communication may at this time be easily established between the receptacles which contain it and any articles of food or drink left unprotected by a suitable covering. At all times when the stools of patients are known or suspected to contain the contagium of disease they should be disinfected in the lavatories before removal—at once when that is possible, or immediately after inspection when inspection is thought necessary. The urine in typhoid and plague should be similarly treated. The matron in a hospital in the East, which is not in communication with sewers by a system of water-closets, should ascertain from time to time that the night soil is being satisfactorily removed; and the nurse in charge of a case of cholera or typhoid in a private house would do well, under similar circumstances, to give the matter some attention.

Native servants need a good deal of supervision in their work, and are especially apt to be careless in matters of the kind under discussion where the importance of small details is not clearly apparent. Disinfectants should be liberally used, and the kitchens kept well supplied with meat safes and covers for the protection of the food there. The native cooks should be instructed as to the importance of keeping food and drink constantly covered.

Water, if at all of doubtful character, should be boiled before being drunk. When water is supplied in a house, a Berkefeldt filter attached to the service pipe is a safeguard and a great convenience. Pressure filters on the Pasteur principle are the only ones which can be trusted.

The adulteration of milk is seldom practised by the native dealers in any other way than by the addition of water, but the water used may be, and often is, of very doubtful purity. In the case of milk, besides the possibility of deliberate adulteration, the quality of the water used to wash the utensils employed in collection and distribution, and the cleanness of the hands, and even the clothing of the milkers, are important considerations, being moreover details not at all likely to receive much attention from the native dairymen. The milk supplied by a native dairy should be boiled as soon as possible after it is received.

(*To be continued.*)

---

*Erratum.*—Under the heading "Beef Juice and Beef Mince," in last week's issue, the time for cooking the mince made from raw meat is wrongly given as an hour instead of half-an-hour. Thirty or thirty-five minutes is the exact time advised in the recipe in Mrs. Elma Stuart's book, entitled "What must I Do to get Well?" from which the details of cooking beef mince are taken.

### Echoes from the Outside World.

AN OPEN LETTER TO A HOSPITAL NURSE.

Amongst the clothing made by the members of the Berkshire and Buckinghamshire Needlework Guild are three articles contributed by the Royal patroness, the Princess Beatrice. She has knitted a warm cot-quilt of pale green and violet wool, with a little black introduced, and also a light-blue baby's woollen jacket, and some pink baby shoes. But there is nothing from the Queen, who seldom fails to send a yearly contribution of some kind. This year, however, such leisure, necessarily very small, as she had to spare for works of charity has been spent in labour of love for her soldiers and forwarded to headquarters direct. Particulars of some of these have found their way into the newspapers; many more have remained unknown to the outside public.

Every country has, of course, a right to its own opinions, and if France thinks highly of Mr. Kruger as a man and a hero, although we cannot admire its taste, it is quite at liberty to express its admiration by bunting and speeches and general enthusiasm. But when once such an excitable people as the French let their sympathies or antipathies get the lead of them, they are little better than maniacs, as their past history has proved over and over again. Therefore, it would have been a matter of everlasting regret if some Parisians, more headstrong than the rest in shouting "A bas l'Angleterre," had put the action to the word and committed an outrage upon English men or women which would have needed an explanation. The French Government and officials were fully alive to the situation, and did their best, whilst allowing Mr. Kruger to make allusions to the British as the "worst barbarians he has ever had to fight, who armed Kaffirs against the Boers, hunted women and children, leaving them without protection, without a roof, and often without bread," to take care that no damage more serious than word-throwing was allowed; and so good was their best that they maintained order throughout the trying crisis. Amongst the comic touches the statement that when in Paris heads were bared, flowers showered into the carriage, and cries of "Vive Kruger," "Vive l'Indépendance" rent the air, Mr. Kruger "smiled pleasantly," struck me as very funny. He could hardly have been expected to "sneer disagreeably" or to "frown heavily," though the handkerchiefs which some demonstrative ladies threw into his vehicle suggested to me the tears he might have shed for the thousands of widows and orphans he has voluntarily made amongst his subjects. Two points about the reception of the ex-President by the French are impressive. First, that had the Paris Exhibition not been over, and English patronage no longer necessary to success, the manifestations of Boer sympathy would have been much less fervid, and the dislike of England much less accentuated; and secondly, that though the wily Dutchman thanks the "French Government for the proofs of interest that quite recently it has been good enough to give," these proofs have been hitherto entirely confined to words; and as long as they do so no great harm will be done.

The death of Sir Arthur Sullivan was sad news for us all. Even those who are unfortunate enough to have "no music in their composition," to use a familiar but rather foolish expression, must in their inner consciousness have felt the appropriateness of the setting of Mr. Gilbert's comic words, though they failed to appreciate the melody of the numbers. But to those who are really fond of music such compositions as "The Golden Legend," "The Lost Chord," "If Doughty Deeds," "The Mikado," and "Patience" have been, and always will be, a source of genuine unalloyed pleasure. It is wonderful that the same brain could produce music of such a totally different character as the hymn tune "Onward, Christian Soldiers" and "Three Little Maids from School," but the mere fact of this versatility probably did much to keep the musician's style so fresh and original. Sir Arthur Sullivan used to say that a great number of his best thoughts came to him whilst he was trying to get to sleep, and "The Lost Chord" in particular was a genuine inspiration, for the tune flashed into his mind whilst he was watching beside the bed of his dying brother Frederic. At his own bedside the son of that brother, whom he adopted, was present; but it was his nurse, who, observing alarming symptoms in the morning of Thursday, despatched a messenger for the doctor. However, before the medical man could arrive the end had come, and it was in the nurse's arms that the patient expired at nine o'clock. The son of a clarionet player, who afterwards became a bandmaster, Sir Arthur's musical genius began very early to show itself, and at the age of eight he belonged to a church choir, and composed an anthem, "By the Waters of Babylon." After Sir George Smart had heard him sing he admitted him to the choir of the Chapel Royal, where, with much fitness, the funeral service (with the Queen's permission) was held on Tuesday, prior to the interment in St. Paul's Cathedral. At fourteen he composed another anthem, "O Israel," which had the honour of being published by Messrs. Novello. He had the advantage of studying under the best masters in England and at Leipzig, and his first great success was the "Tempest" music. From then almost to the end his career was a series of musical triumphs.

When the electric light was first introduced to the public one of the points in its favour was said to be that it avoided the risk of fire which accompanied the use of gas and paraffin oil. But, according to recent events, the fear of conflagration is as great with electricity as with any other illuminating power. A current, which is in itself quite harmless, may through certain defects become highly dangerous, and only last week a serious outbreak of fire took place which the fire brigade attributed to the fusing of the cables at the switch-board. What is called "earthing" is the great element of danger, but then experts are at present unable always to prevent the accident of earthing, and considering that electricity is being so largely employed in churches, hotels, and hospitals, where, especially with regard to the latter, the avoidance of fire is so important, the matter is one which needs attention. It is believed that many of the accidents might be avoided if the regulations as to installation were more stringent than they are at present; and it is to be hoped that the representations on the subject which it is proposed to make to the Board of Trade may result in a more satisfactory state of affairs. If not, electricity as the means of giving domestic light will fall into disrepute.

Those of you who are admirers of Mr. George Alexander—and they are legion—will probably like to see him in one of his old parts at St. James's Theatre. I do not mean that he is playing for the second time a rôle in which he scored a success before, but that, instead of being a man with a past which ruins his present and his future, or a man of forty too jaded to believe in anything or anybody, he is once more the young lover whose love makes him happy and healthy. The Duke of St. Asaph, though a married man, is only a Benedict of a fortnight, and is as much in love with his wife as he well can be. That he does not thoroughly confide in her when he goes to act as a peacemaker is, apparently, because Mrs. Craigie has a story to tell, not because he has anything of a compromising character to hide. On the contrary, "The Wisdom of the Wise" is refreshingly pure and sweet all through, though the society dames whom "John Oliver Hobbes" loves to depict and to caricature, do their best to unearth a mare's nest. Miss Fay Davis, as the young Duchess, is, of course, very charming, and her first dress of Sèvres-blue satin, sparkling with diamonds, will rouse every woman's admiration. Miss Julie Opp looks strikingly handsome, and all the characters are entrusted to actors and actresses who can make them everything that they should be. The conversation is as brilliant and as amusing as readers of Mrs. Craigie's novels and listeners to "The Ambassador" would naturally expect, but not to my mind more clever than Mr. Alexander's closing words at the fall of the curtain when, after he had thanked the ladies and gentlemen among the audience who seemed to like the play, he referred to a group of dissentients in the gallery as the inevitable, because, "As there is no rose without a thorn, so there is no first night without a 'boo.'"

### The Crisis at Croydon Infirmary.

By Our Own Correspondent.

THE strained relations between the Croydon Guardians and the matron of the Infirmary were again the subject of debate at a meeting of the Board on Tuesday. It will be recollected that the matron refused to sign the certificates of three probationers at the close of their term of training on the ground that they were incompetent. The Guardians were advised differently and passed resolutions taking the charge of the nurses away from the matron. Miss Julian thereupon appealed to the Local Government Board, and over twenty of the probationers also memorialised against the change, stating that certificates of training without the matron's signature were in the nursing world practically useless. The Local Government Board asked the Croydon Guardians to reconsider their decision, drawing special attention to the petition of the probationers. This letter was referred to the Infirmary Committee for report.

On Tuesday the matter was fully discussed. As part of the new arrangement the Guardians at a recent meeting assigned fresh duties to Miss Sheppard, the assistant matron, with an increase of salary. The Local Government Board wrote that the new proposal with regard to Miss Sheppard would be considered in connection with the matter concerning the matron.

Then the Board proceeded to the main question.

The Infirmary Committee reported that they had considered the letter of the Local Government Board of the 16th inst. relative to the questions which had arisen in connection with Miss Julian, the matron of the Infirmary, and they recommended that the following observations be furnished to the Local Government Board in reply thereto, namely:—

(1) That the Guardians are unable to understand the expression in the Board's letter that "the Board are not in possession of sufficient information to enable them to express any opinion as to the course adopted by the Guardians," inasmuch as the Guardians have not themselves appealed to the Local Government Board on the subject of the controversy which has arisen between them and the Matron of the Infirmary. The only communication which the Guardians have addressed to the Board on the matter is the one which the Clerk was directed to forward on the 17th ult., furnishing the observations of the Guardians on the subject of Miss Julian's communication to the Board of the 25th September last, which was duly addressed to the Local Government Board in response to their request of the 5th ult.

(2) That the Guardians join with the Local Government Board in regretting the state of things indicated by the correspondence which has taken place on the subject referred to, and are fully alive to the importance of adopting suitable arrangements for the supervision of the nursing staff and nursing duties, as well as for the training of the Probationers, and that they have already made tentative arrangements with this object in view, and will shortly submit to the Board a proposal with respect to the appointment of a Superintendent of Nurses.

(3) That the petition to which reference is made was fully considered by the Infirmary Visiting Committee on September 25th last, and a deputation of the Nurses was interviewed by them on the subject, but as there was evidence that the action of the Nurses was instigated by the Matron it was not deemed necessary to make any recommendation to the Guardians on the subject.

(4) That the Guardians again emphasise the fact that they do not apprehend, neither do they think it possible, that the resolutions adopted by them will have the prejudicial effects referred to by Miss Julian, and that the Guardians are quite prepared to take the responsibility of their action in the matter.

The Committee recommended the Board to assent to the necessary steps being taken by the Committee with a view to the appointment of a Superintendent of the Nurses.

Mr. SHIRLEY moved the adoption of the report. What the Guardians had done, he said, was perfectly in order. When the Infirmary was first opened it was for the care of the sick and needy, and there was no idea in those days of making it in any shape or form a nursing institution. The system of probationers had worked very well, but at the same time the Infirmary Committee must have power to manage its own affairs. They could not have their work upset by the young people, but must make a stand. He now asked the Board to sanction the reply to the Local Government Board as set out. Referring to the recommendation to appoint a superintendent of nurses, he asked that the Committee be allowed to advertise for a person who would be able to take charge of the young people and give them lectures as before.

Mr. T. P. WOOD seconded.

The CHAIRMAN referred to alleged misrepresentations and errors in the press as to the misunderstanding which had arisen, and in order to show what had really taken place, read extracts from correspondence covering the whole period.

On July 10th Dr. Wilson, Medical Superintendent of the Infirmary, forwarded to the Board five Probationers' certificates for sealing and signing, but stating that the Matron had refused to sign three of those. The Board referred this refusal to the Special Infirmary Committee. On July 17th this Committee reported that they had considered this refusal, but the Matron being on leave they could not interview her. The Medical Superintendent however reported to the Committee that the Matron said she refused to sign as the Probationers were insolent. The Committee recommended that Miss Julian, on her return, be requested to sign the certificates in their present form. On August 2nd the Clerk wrote to Miss Julian on behalf of the Board, requesting her to be good enough to sign the certificate immediately on her return from leave of absence. On August 4th the Matron replied, "I regret I cannot comply with your request, and will give my reasons for not doing so to the Board if required. On September 11th Miss Julian wrote to the Board: "I have refused to sign the certificate. (1) It is generally held that the bestowal of a certificate on a nurse on the condition of her training is a guarantee to the nursing profession and to the public that the nurse is, in the opinion of the authorities of the institution which has trained her, fully qualified to take her place in the ranks of graduate nurses, and to practise the duties of her calling. (2) In my opinion, these women are not so qualified. They are uncultured, most selfish, and entirely lack the deep sympathy with suffering and that devotion to duty which marks every true nurse, and which is essential in every woman who ministers to the sick. (3) For me, a trained nurse and hospital matron, to certify that the conduct of these three women has been satisfactory, and that they are conscientious, reliable nurses would be, in my opinion, equivalent to perpetrating a public fraud, and this I decline to do." On September 20 the Board wrote to Miss Julian, saying that they were unable to approve of her action, and had decided that the signature of the Matron of the Infirmary on the training certificate of Probationer nurses be dispensed with.

The CHAIRMAN, having read all these extracts, observed that the public would see, he thought, that there was nothing against these nurses to damage them. The Local Government Board in their letter were certainly sitting on the fence, and he thought that Board should have either held an inquiry or upheld the Guardians' action. The Local Government Board evidently did not know what to do, and were afraid of their own opinion. He did not think it was the correct thing for the Local Government Board to send the evasive reply which they did. With regard to the recommendation of the Committee as to a superintendent of nurses, he could say plainly that it had nothing to do with the letter of the Local Government Board as the committee had intended to take action quite independently. He thought after the press and the public had digested the manner in which the Guardians had acted, they would uphold their action and consider that they were perfectly right in what they did. He was quite certain that no public body having any respect for itself could have done otherwise.

Mr. WUSTEMANN said a short time ago Mr. Shirley complimented Miss Julian on the way in which the probationers got on. He should most certainly vote against the motion, and admired Miss Julian's spirit in standing up for her rights.

Mr. SIBUN was very sorry to go against the Committee. He objected to the introduction of a third officer. If probationers did not like to come knowing that their certificate would be signed by the medical superintendent let them keep away.

Mr. SHIRLEY said on no occasion during the three years had Miss Julian found fault with these probationers.

Mr. SIBUN moved an amendment in opposition to the new appointment.

The Rev. R. A. BOYLE said if they adopted the amendment they would be knuckling under to Miss Julian. If there were any signs of change in Miss Julian's spirit he felt quite sure that every member of the Board would be only too anxious to let bye-gones be bye-gones. It would be false economy to adopt the amendment. If institutions like their infirmary ceased to be institutions for training young nurses where would the supply be in a few years? He did not think that the Board would upset a system that had worked so well, and would have continued to have worked well but for the impracticable attitude adopted by the matron.

Mr. OWEN thought the action of the Committee premature pending the decision of the Local Government Board. What position would the Board be in if, having proceeded to appoint a superintendent of nurses, the Local Government Board came down and said they had no right to depose Miss Julian?

Mr. MORLAND said they were bound by their position to go forward with regard to the necessity for the appointment. If they had a nursing school at all they must have someone to give the necessary instruction.

Mr. ELSLEY supported the amendment. There was not the slightest occasion for a superintendent of nurses. They had in Miss Julian a very efficient nurse in every way, and from what they could learn that afternoon no one could say anything against her as to her teaching of the nurses.

Mr. SHIRLEY, in closing the debate, hoped the Board would not support the amendment. So far as the probationers were concerned, it was impossible for the medical superintendent to train them in detail. Replying to Mr. Elsley, he could not agree with him as to Miss Julian. It was a complete answer that she had declined to carry out the instructions of the Board. If they gave up the system of training probationers it would mean an outlay of £800 a year.

There voted for the amendment not to appoint a superintendent of nurses . . . . 10
For adopting the report . . . . . . 18

The report was therefore agreed to.

### Minor Appointments.

ROTHERHAM HOSPITAL.—Miss Gertrude Wilkinson has been appointed night sister. She was trained for three years at the Wolverhampton and Staffordshire General Hospital.

CLAYTON HOSPITAL, WAKEFIELD.—Miss Ada Mary Smith has been appointed sister of the children's ward. She received three years' training at the same institution, and remained a fourth year as staff nurse. She has since taken the diploma for midwifery at the Royal Maternity Hospital, Edinburgh, and also done some private nursing.

BRADFORD CITY FEVER HOSPITAL.—Miss Ethel Dickinson has been appointed charge nurse. She was trained at Norwich Fever Hospital, and has since been assistant nurse in the same institution; assistant nurse Parkhill Fever Hospital, Liverpool; assistant nurse Middlesbro' Smallpox Hospital; and assistant nurse City Fever Hospital, Bradford.

### To Nurses.

WE invite contributions from any of our readers, and shall be glad to pay for "Notes on News from the Nursing World," or for articles describing nursing experiences, or dealing with any nursing question from an original point of view. The minimum payment for contributions is 5s., but we welcome interesting contributions of a column, or a page, in length. It may be added that notices of entertainments, presentations, and deaths are not paid for, but, of course, we are always glad to receive them. All rejected manuscripts are returned in due course, and all payments for manuscripts used are made as early as possible at the beginning of each quarter.

### Everybody's Opinion.

[Correspondence on all subjects is invited, but we cannot in any way be responsible for the opinions expressed by our correspondents. No communication can be entertained if the name and address of the correspondent is not given, as a guarantee of good faith but not necessarily for publication, or unless one side of the paper only is written on.]

### ASYLUMS BOARD NURSES.

"GRATITUDE" writes: I shall feel obliged if you can find space in NURSING MIRROR for a few words on the subject mentioned by "A Nurse in the Colonies." Your correspondent speaks of some of the advantages enjoyed by nurses working under the Metropolitan Asylums Board, particularly the good salary given, off-duty time allowed, &c., but there is one other provision made, which is not always appreciated as it should be, and that is the great care and unceasing kindness shown towards those nurses who are sick. Personally, I feel I cannot sufficiently express my gratitude for the many little attentions shown me, both by the nurses in attendance, and also by the matron and the doctors, during my recent illness, which I unfortunately contracted in the discharge of my duties. Of the nurses I cannot speak too highly, for their untiring devotion towards their incapacitated fellow-workers, and the matron's daily visits to which we all looked forward, and the constant medical attention, deserve the highest praise. The matron, especially, does many "little kindnesses which most leave undone," and I think it only right that it should be mentioned. Much more might be said about our many privileges (so often overlooked, or taken as a matter of course), but I mention these because I feel that in doing so I am expressing the gratitude and thanks of many more besides myself towards those who so willingly, unselfishly, and untiringly devote their lives to a splendid work of love and service.

### NURSE DOWLING'S ASSERTIONS.

"A. P." writes: The case of Nurse Dowling to me is a somewhat pathetic one, and her training has certainly been a most unfortunate and trying one, and I can surely say for my fellow-nurses we are all very sorry for her. For my own part, although speaking with small experience, a nurse's life is a sacrifice: we lay down our lives for our patients, and not our matrons, and when we are in the sculleries doing work which we were never used to, perhaps we can think of those who are sick, helpless, and dying in our wards, and thus make it easier. We live and work for them alone, and surely Nurse Dowling must have lived for her own comfort. We should be more encouraged, of course, if those over us could and would make it easier, and remember that they too had their probationary time and were glad of both rest and encouragement, change of scene and air, for we do get very depressed. We should be glad of our off-duty days as they are due to us, for we need them and they are few. Our salaries are also small, and I think that if the large sums of money left to hospitals were shared by nurses as well as others it would be better expended, for we spare hospitals many wardmaids and porters.

"M. L. F." writes:—It seems to me that Nurse Dowling is one of those women who never see the bright side of anything. According to her idea, neither matrons, doctors, judges, nor patients, do the right thing. And I cannot imagine how she has remained so long in the nursing world as eight years. It would have been better for her patients if she retired, one would think. I have been nursing much longer than eight years, and was trained at the "London," and I can truthfully say that some of the happiest days of my life were spent in hospital. As to being happy or miserable, in my opinion it rests entirely with the nurse herself. There are not many who give up nursing when once they take to it in earnest, for it is a fascinating and happy life to those who choose to make it so. However, I do not think there is any fear that intending probationers will take Nurse Dowling's advice after reading her letter. It is absurd to talk of matrons sitting in easy-chairs and reading novels. I for one should be glad to think they had the opportunity to do so occasionally.

"NURSE DOROTHY" writes: I cannot quite agree with Nurse Dowling. Her letter is too severe and very unjust, I

think. *Re* hospital duties, a probationer does find it hard and difficult at times to please those who are superior to her in hospital life and to those of her fellow-nurses. Still, I do think there is much pleasure and happiness in the doing of ward duties if a pro. goes to work with a Christian spirit, a love for her work and a determination to overcome all difficulties. No pro. was more unhappy than myself when first entering upon hospital duties; no pro. felt her duties more hard and severe; but at the end of my training no old pro. could be more sorry to leave her fellow-nurses and hospital duties than I was. Therefore I say the remarks passed on our esteemed matrons and doctors are very uncalled for, Nurse Dowling asserts that: "There is no pity for a nurse when she is ill, or when she has just returned from a death-bed." We as pro.'s do find it a little hard when feeling tired and worn out; but what of those friends whose loved ones we have just seen pass from this life? We should most entirely forget ourselves then, and give all the love and sympathy we can to those whose loss is keen indeed. With regard to private work, speaking from my own experience, I have at all times met with every kindness and consideration. As a Christian woman and a fellow-worker perhaps Nurse Dowling will pardon my saying it, but I think very much depends on the nurse herself.

"PHILOSOPHER" writes: Nurses everywhere ought to raise their voices unanimously in protest against the very far-fetched assertions of Miss Dowling concerning hospitals and matrons. She, it would appear, had an exceptionally hard time; but why attack all nursing institutions and their matrons for the shortcomings and ill-doings of a few individuals? Nurses are no more overworked than any other class of workers in this rush-along century. Indeed, the majority of nurses are much better housed and better fed than the vast numbers of young people in the various business houses and offices, &c. In every position in life there is much that is unpleasant to put up with; in every life that one can lead there is much that is petty and annoying to meet with every day. But nurses fare no worse than other people. They are generally treated with more kindness and consideration by those in authority over them than are the "young ladies" who, however weary, have to look agreeable under the shop-walker's eye in even the best-class shops. It is nothing but absolute nonsense, if not worse, to say that because we have occasionally some unpleasantnesses our lives become burdens to us. We deserve admiration, if so, for bearing them so remarkably cheerfully! While, as women, we all sympathise with the hardships Miss Dowling has undergone, as nurses we sincerely regret that she should make such injurious and unmerited accusations against the whole nursing world, for nursing institutions, though they may not be perfect, are, in this country at all events, daily reaching a higher standard.

"K. E. B." writes: I should like to give a reverse side to the public to that given by Miss Agnes Dowling. I am a nurse of sixteen years' experience, who was trained in a general hospital, and have since served under the administration of other institutions, including private and district nursing homes; in fact, I have filled every post from probationer to matron—the latter for some years. Though I had as a probationer to conform to strict discipline, and do plenty of hard work, my duties could not be truthfully compared to a treadmill. And looking back to that time, I have only one feeling—namely, that of gratitude for the just way in which the method of training was conducted. Then as a private nurse I always found patients, doctors, and the superintendents of the nursing homes treated me with kindness and consideration. There was always a warm welcome when I came in tired from a case. As a district nurse my experience was one of loving gratitude from patients and appreciation from those placed over me. I was never made to feel like a burden either by being underfed, or underpaid, or by unkind treatment. I have had less time to read novels since I became a matron than I had when I was a nurse. There are some nurses who would always be discontented and ready to suggest systems of reform in institutions. They are the people who would do well to exercise more self-discipline. They are always dwelling on their own fancied, or it may be real, grievances, with the result that they grow selfish and their work suffers. If they thought a little more of the real suffering it is their mission to soothe, they would gain that self-forgetfulness which would bring in due course a harvest of affection and loving sympathy in their work and the discomforts that are inevitable to a nurse's calling.

"LOYALIST" writes: I cannot refrain from writing in protest against Miss Dowling's letter. I should have thought that eight years of nursing whould at least have taught her loyalty. How much that word means and how little it is encouraged! Having been connected with at least six institutions is a fact in itself to prove that the fault must lie in Nurse Dowling herself, especially when she adds that nearly all patients and friends of patients refused her assistance when she was in trouble. But some people expect so much—a little work, food of the choicest, large salaries, and the burden of life taken from their shoulders. Hospital life to me as a probationer was certainly not a treadmill, although those of us who are older had not the privileges of the probationer of to-day. Why, I ask, should a matron not sit in an easy-chair, or read a novel? Nurses frequently have the same opportunity, and, moreover, a nurse generally knows when she is off duty that she is free, whereas a matron never, unless she is actually away from the hospital. Oh! to think that a nurse can say "there is no pity for her when she is ill." Perhaps the nature of Nurse Dowling's illnesses required very firm treatment which she mistook for lack of sympathy. For myself I have received unremitting kindness from everyone when ill. Nurse Dowling cannot complain of being refused a testimonial after two years' work, for she appears to have understood the rule that one would not be given. The advice which she gives to intending probationers might with advantage have been taken by herself years ago, and I do not think that the profession would have suffered thereby. How gladly would many of us when serving on the private staff have gone back into the hospital for a time! And it is with affection and respect that I remember my old matrons who have now retired and are enjoying their much-earned rest after years of labour—especially one who was ever ready to help and guide any who sought her advice, whether in matters professional or private, so wise, so tactful, and so motherly. I refer to the late matron of Westminster Hospital. And we who are matrons now look back with thankfulness that we were privileged to be trained by such.

### Appointments.

SAVERNAKE HOSPITAL, MARLBOROUGH.—Miss Vesey has been appointed matron. She was trained at St. Thomas's Hospital, where she has since been sister.

ROCHDALE CORPORATION INFECTIOUS DISEASES HOSPITAL.—Miss Isabel Kewley has been appointed matron. She was trained at Salop Infirmary, Shrewsbury, and has since held appointments at Warrington, St. Helens, and at the Borough Sanatorium, Huddersfield.

SMALLWOOD HOSPITAL, REDDITCH.—Miss Mary Barwick has been appointed matron. She was trained at Leeds General Infirmary, and has since been nurse matron at Bridgend Cottage Hospital, and matron at the Haverfordwest and Pembrokeshire Infirmary.

VICTORIA CENTRAL HOSPITAL, LISCARD, CHESHIRE. — Miss G. B. J. Bailie has been appointed matron. She was trained at the Northern Hospital, Liverpool, and has since been sister at the Infirmary, Blackburn; sister at the South Devon and East Cornwall Hospital, Plymouth; and for the last three years matron at the Cottage Hospital, Seacombe.

BARTON REGIS INFIRMARY, BRISTOL.—Miss Jean Gordon has been appointed superintendent nurse. She was trained for three years at Bristol General Hospital, and she has since been two years on the private nursing staff, three years in charge of the midwifery department, and for the past year sister of two of the large medical female wards. Miss Gordon holds the L.O.S. certificate.

### For Reading to the Sick.

"YE shall leave me alone; and yet I am not alone, because the Father is with me."—*St. John* xvi., 32.

MIDNIGHT HYMN.

In the mid silence of the voiceless night,

When chased by airy dreams, the slumbers flee,

Whom, in the darkness, doth my spirit seek,

O God, but Thee?

And if there be a weight upon my breast,
Some vague impression of the day foregone,
Scarce knowing what it is, I fly to Thee,
And lay it down.

More tranquil than the stillness of the night,
More peaceful than the silence of that hour,
More blest than anything, my bosom lies
Beneath Thy power.

For what is there on earth that I desire,
Of all that it can give or take from me?
Or whom in Heaven doth my spirit seek,
O God, but Thee?

*Author Unknown.*

MS. found in a Cottage in England.

It is not always in the pleasant pastures and beside the still waters of life that we are led by the Shepherd of our souls to rest; perhaps it may be ours to find it in some silent vale of penitence and tears; sometimes in sore weakness of body, sometimes in lonely watching, sometimes in days of slow-declining health, sometimes in the busiest whirl of active work for Christ, we find our blessed Rest.

Seek neither rest of body nor rest of heart in earthly joys or hopes or consolations, howsoever dear. Rest is in Christ. His worship is the spirit's true repose; His service is our most refreshing toil; His Church, His kingdom, the soul's only home, and our one perfect haven of repose and peace.

God rested on the seventh day, we read, when He found all things "very good"; and still He rests in us, and we in Him, when we are striving to fulfil His word; our souls in tune with Him, our hearts obedient to the tender guiding of His holy will. And He is waiting now, with patient hands outstretched to draw us to Him for eternity, that we may be at rest indeed, and at peace with Him; our work on earth all done by His enabling grace, and by His master eye reviewed and blessed as "very good."—*E. A. D.*

ALONE WITH GOD.

*St. Mark*, i. 35.

O Thou, Who rising long before the day,
Went'st forth to pray
On the cold mount, by weariness oppressed,
That we might rest
With Thee hereafter; though my lot denies
Sleep to mine eyes—
Blessed Redeemer, how can I repine,
Remembering Thine?

O Thou, Who at the fourth watch of the night
Didst come in sight
Of Thine Apostles, toiling on the wave;
And, swift to save
From peril and from fear—saidst, drawing nigh,
"Peace! it is I;"—
O still my thoughts, tempestuous as that sea!
Speak peace to me!

O give Thy servant patience, to be still
And bear Thy will;
Courage to wholly venture on the arm
That will not harm;
The wisdom that will never let me stray
Out of my way;
The love that now afflicting, knowest best
When I should rest.

*J. M. Neale.*

### Notes and Queries.

**The Editor is always willing to answer in this column, without any fee, all reasonable questions, as soon as possible.**

**But the following rules must be carefully observed:—**

**1. Every communication must be accompanied by the name and address of the writer.**

**2. The question must always bear upon nursing, directly or indirectly.**

**If an answer is required by letter a fee of half-a-crown must be enclosed with the note containing the inquiry.**

*Children's Nurses.*

ANSWER TO R. C. (70).—Children's nurses, we are informed, can be trained at the Children's Hospital, Temple Street, Dublin. There are 90 beds. The training has hitherto only been for a year, but after this month it will probably be extended to two years, and eventually, it is hoped, to three.

*A Legal Point.*

(89) Three years ago I became a probationer in a union infirmary and signed a printed form in which it was stated that I "should be required to give one month for trial, receiving no salary, but that the month should be counted in my three year's training." I am engaged for a post at the expiration of my three years, but the matron of the infirmary informs me that the guardians expect the nurses to forfeit that month altogether, and I shall be required to remain another month. The Clerk of the Guardians has threatened to keep back our certificates if we do not. 1. Can the clerk, in face of the printed form, legally keep back our certificates? 2. Is there any way of compelling the authorities to grant my certificate and set me free at the end of the three years from date of entry? I have received testimonials from the medical officer and matron, which enabled me to obtain the post I shall probably lose if I am obliged to stay another month.—*Dilemma.*

This is a purely legal question, and the answer depends on the form and wording of the agreement. It will probably be found that there was no agreement at all, and that what was signed was a purely one-sided undertaking. Consult a solicitor.

*Certificates.*

(90) 1. Would you kindly tell me if a nurse's certificate without a matron's signature is of the same value as one with it? 2. Also if there are any training schools granting three years' certificates where the matron does not sign the certificate?—*H.S.W.*

1. A matron's testimony to a nurse's qualifications and character is very important, consequently a certificate without her signature cannot be of the same service as if it were properly signed.

*Consumption.*

(91) Nurse E. C. asks for particulars of a much advertised special "treatment" for tuberculosis. Now Nurse E. C. should first of all remember that treatment is the work of the doctor and not of the nurse, and next she should bear in mind that advertised forms of treatment are almost without exception founded upon quackery and imposition. Surely, it speaks ill for a nurse's training that, at the end of three years in a hospital, she should still be so taken in by advertising quack as to write for information about his "treatment," as if it were a thing to be seriously discussed by reasonable people.

*Arnold's Apparatus.*

(92) W. E. U. wishes to ask what the Arnold knee apparatus is like; if it is expensive; and where it can be obtained?

Again we would warn nurses that treatment is outside their province. The suggestion of any such apparatus should come from the doctor, not the nurse

*Action of the Skin.*

(93) Is there any drug used externally with friction or massage that would cause *perspiration?*—*E.M.K.*

Massage itself tends to produce action of the skin. It is doubtful if any drug used in this process has much effect in this direction.

*Cape Colony.*

(94) 1. Can you give me any particulars as regards nursing at the Cape at present? Should I be likely to get a post on arriving there? 2. And is there anything particular in the way of outfit advisable?—*E. W.*

It is difficult to obtain reliable information in any department so long as affairs are unsettled. You could write to the Lady Superintendent of the Victoria Nurses' Institute, Hoff Platz, Cape Town, and to the Secretary of the Emigrant Information Office, 31 Broadway, Westminster, where you can always obtain the latest particulars. 2. The outfit should be one suitable for hot summer weather, with a supply of thick under flannels, a warm dressing-gown, and a warm jacket. The nights are often very cold.

*Child's Nurse.*

(95) Would you kindly give me name of the institution in London where children's nurses are trained?—*W. B.*

Apply to the secretary, the Norland Institute, 29 Holland Park Avenue, London, W.

*Paris.*

(96) Would you kindly state if there is a good English hospital in Paris; and if a nurse trained in a London Poor Law infirmary is eligible for a post in it?—*Marion B.* and *Bertha.*

The Hertford British Hospital, Paris, Rue de Villiers, Levallois-Perret, Paris, with 34 beds. Apply to the Lady Superintendent for information as regards nursing arrangements.

**Standard Books of Reference.**

"The Nursing Profession: How and Where to Train." 2s. net; post free, 2s. 4d.
"The Nurses' Dictionary of Medical Terms." 2s.
"Burdett's Series of Nursing Text-Books." 1s. each.
"A Handbook for Nurses." (Illustrated.) 5s.
"Nursing: Its Theory and Practice." New Edition. 3s. 6d.
"Helps in Sickness and to Health." Fifteenth Thousand.
"The Physiological Feeding of Infants." 1s.
"The Physiological Nursery Chart." 1s.; post free. 1s. 3d.
"Hospital Expenditure: The Commissariat." 2s. 6d.
All these are published by THE SCIENTIFIC PRESS, Ltd., and may be obtained through any bookseller or direct from the publishers, 28 and 29 Southampton Street, London, W.C.

### Travel Notes.

By Our Travelling Correspondent.

### LXII.—SIX MONTHS IN ROME.

I hope to write a few papers on Rome and the cities of Northern Italy this winter in reply to some kindly appreciative letters that I have received expressing the wish that I would go further afield in our mutual wanderings, and be a little more diffuse in my accounts. It is always very gratifying to receive such communications, and to feel that one has succeeded in interesting one's readers. Thus encouraged by my kindly public I also purpose from time to time to give short accounts of art on the Continent as met with in painting, sculpture, and architecture, in hopes that when my readers encounter these priceless treasures of the past they may seem almost like old friends. I am doubtful whether such articles will be of very general interest, therefore they will only appear occasionally, lest I weary those friends whose tastes lie in totally different directions. I have headed this article "Six Months in Rome" (though my own experiences there extended over two years) because I think anyone with management may contrive to see everything of primary importance in that time, though the real spirit of Rome takes long to sink into one's inner self.

### First Impressions.

It is grievous, but true, that one's earliest feeling is disappointment—I think every thoughtful person experiences the same sensation; probably the reason is that we have had magnificent expectations, and time is needed to adjust proportions. Rome is not to be seen by the passing tourist in a month, or still less in the really admirable ten-guinea trip advertised by Dr. Lunn, which gives six days in Rome. No; it must be studied leisurely and with discrimination, not bolted *en masse*.

Far be it from me to suggest that six days in Rome is useless. I have such a strong opinion as to the mental and moral bracing effect of travel that I should certainly say six days in the Eternal City is far better than none at all—*Cela va sans le dire*—but if you are fortunate in purse and leisure, a long stay will teach you to feel and love its strange fascination and long ever to return to its embrace. Howells has well expressed this feeling in his "Roman Pearls"—

"And, alas! there I caught the Roman fever, the longing that burns one who has once been in Rome to go again—that will not be cured by all the cool, contemptuous things he may think or say of the Eternal City; that fills him with fond memories of its fascination and makes it for ever desired."

### Plan of Sight-Seeing.

I think my own plan of studying the sights as revealed to me by my journal is as good as any we can adopt. I thought my residence in Rome would be limited to three months, so that everything was considered with regard to that restriction. I shall say but little of ancient Rome, the subject is too vast and too learned for our humble little talks together, and there are such excellent books on the subject written by earnest students and open now to all, that I shall confine myself to pointing out the charm existing for us modern folk in a close acquaintance with mediæval Rome; the bonds, however, which link this period on the one side to ancient, and on the other to modern Rome are too firm to be disunited.

### The Pincio.

First, then, let us go to the Pincio where one receives an impression never effaced. Ouida gives an excellent description of it in *Friendship*.

"It was sunset on the Pincio. . . . Beyond St. Peter's there was that sky of purple and of gold, which always seems so much more marvellous here than it does anywhere else; that rose-leaf warmth and soft transparency of flame-like colour which those who have looked on it never will forget, so long as their lives shall last. . . . Below, loud, cracked, discordant bells were chiming one against another; near at hand a military band was playing very fast and very much out of tune waltzes of Strauss; but nothing spoiled the grandeur of the scene, or could destroy the sublime calmness of declining day, as the broken green lines of the hills grew black against the burning scarlet of the clouds, and the vast expanse of roofs and spires, cupolas and towers, obelisks and gardens, ruins and palaces, colossal temples and desolate marshes that is called Rome, stretched away wide and vague and solemn as a desert; with a sun nearly as red and rayless as the desert's, hanging above the cross on the great Dome. It was four o'clock and there was the customary crowd of fashionable idlers, fretting horses, emblazoned carriages, sauntering dandies, handsome artists, tired invalids, black-robed priests and scarlet-clad janitors, cuirassed soldiers and curly-headed children, violet-gowned seminarists and purple-gowned scholars, and first and foremost fashionable ladies chattering at the top of their voices about the first foxhunt of the year, the first Court ball, the new arrivals, and the Pope's state of health.

"The sun was going down in majesty behind the round domes raised to lay the restless soul of Nero; but up here on the hill nobody looked at it, but idling, laughing, and talking turned their backs to the west to hear the music better. . . ."

No words of mine could give so vivid a description of the Pincio as the gifted novelist's, and they were in my mind when I, too, swelled the crowd on that small but cosmopolitan promenade, and looked with wonder and awe over the city so full of the romance—the tragedy—the blood-guiltiness and the enormous power for good and evil of the past.

To turn to lighter thoughts, my heart went out very much to the Bersaglieri, garrisoning Rome at that time in conjunction with artillery and cavalry. One officer in especial was a joy to me; he admired himself so much that he could hardly "put his foot to the ground for delicateness"! but then he was a very beautiful person in his black and cherry colour and his entrancing picture hat with flowing plumes. He must, I think, have been to some grand function, for the officers seldom wear their sombreros, but merely an ordinary képi. With him was a cavalry companion, a "dear little or'f'cer boy" young and callow, but who wore his lovely sky-blue cloak with native grace.

These sons of Mars held divided empire in my heart with certain gorgeous students from the German college, very brave in scarlet, which set off their round blonde Teuton faces to perfection. I loved to see them in the twilight lighting up the darkening shadows in the sombre old streets.

### TRAVEL NOTES AND QUERIES.

Rules in Regard to Correspondence for this Section.—All questioners must use a pseudonym for publication, but the communication must also bear the writer's own name and address as well, which will be regarded as confidential. All such communications to be addressed "Travel Editor, 'Nursing Mirror,' 28 Southampton Street, Strand." No charge will be made for inserting and answering questions in the inquiry column, and all will be answered in rotation as space permits. If an answer by letter is required, a stamped and addressed envelope must be enclosed, together with 2s. 6d., which fee will be devoted to the objects of the "'Hospital' Convalescent Fund." Any inquiries reaching the office after Monday cannot be answered in "The Mirror" of the current week."

Rouen or Avignon (Undecided).—It is a curious alternative, but I should not hesitate a moment. Avignon is full of interest, is much warmer in climate, and is surrounded with such a wealth of interest in scenery and splendid cities of the past, as to afford study for years. Nimes, Arles, Tarascon, and Orange are all within easy distance, and think of the surrounding country. You can visit Rouen any summer in a week's holiday. Dress much as in England, all woollen clothes, but of varying thicknesses. No, not at all gay.

Cathedrals in N. France (Gothic).—In a fortnight you could see the following cathedrals easily:—Amiens, Beauvais, one night in Paris to see Notre Dame, Chartres, Evreux, Rouen, and Caen. Go viâ Boulogne and return by Caen. If you make out this tour, Messrs. Gaze or Cook can probably arrange it for you. It is too much to do in the time really, but that is a matter for your own consideration. Remember, the trains are rather slow.

